# Nutritional information on the labels of processed and ultra-processed foods and beverages marketed in a supermarket chain in Lima in 2022

**DOI:** 10.17843/rpmesp.2023.402.12714

**Published:** 2023-06-30

**Authors:** Mayra Meza-Hernández, Kiomi Yabiku-Soto, Lorena Saavedra-Garcia, Francisco Diez-Canseco

**Affiliations:** 1 CRONICAS Centro de Excelencia en Enfermedades Crónicas, Universidad Peruana Cayetano Heredia, Lima, Peru. Universidad Peruana Cayetano Heredia CRONICAS Centro de Excelencia en Enfermedades Crónicas Universidad Peruana Cayetano Heredia Lima Peru

**Keywords:** Nutritional Labeling, Nutritional Facts, Industrialized Foods, Peru

## Abstract

**Objectives.:**

To estimate the number of processed and ultra-processed beverages and foods that provide nutritional information on their packaging, and to describe the characteristics of this information, as well as to determine the presence of nutritional information on products with octagons.

**Materials and methods.:**

Photographs were taken of the labels of 4404 processed and ultra-processed beverages and foods marketed in supermarkets in Metropolitan Lima. The information on the label was collected and registered in the mobile and web version of the Food Label Information Program (FLIP). We analyzed variables related to the nutritional information, the way in which such information is declared and the information in beverages and foods with octagons.

**Results.:**

Only 71.4% of the products had some type of nutritional information. Of these, 13.8% provided the nutritional information as a text and not in a table, and only 56.3% declared it per 100 grams or milliliters. Of the total number of foods with the octagon “Contains trans fats”, only 19.2% declared their content.

**Conclusions.:**

More than a quarter of the beverages and packaged foods in the Peruvian market did not provide nutritional information of any kind, and of those that did, only one did so in different formats and units. In addition, we found that a proportion of beverages and foods for each type of octagon did not declare information of the nutrient that is mentioned in the octagon.

## INTRODUCTION

In recent years, the consumption of processed and ultra-processed foods and beverages has increased worldwide ^1^. These types of products are characterized by high amounts of critical nutrients (saturated fats, sugar, sodium and trans fats), whose excess consumption is a risk factor for developing chronic noncommunicable diseases ^2^. In Peru, sales of ultra-processed foods increased by 8.9% between 2009 and 2014 (from 294 to 320 kcal per capita/day), while those of ultra-processed beverages increased by 6.7% (from 114 to 121 kcal per capita/day) ^1^.

In response to this situation, the Peruvian government established policies to promote healthy eating ^3^^-^^5^. One of these policies is the Law for the Promotion of Healthy Eating in Children and Adolescents (Law No. 30021) ^3^. This law states that processed and ultra-processed foods and beverages that exceed the established parameters for critical nutrients must carry a frontal advertising warning in the form of black octagons (hereinafter “octagons”). These octagons indicate that the products are high in sodium, sugar and/or saturated fat, or that they contain trans fats ^6^. However, the exact content of these nutrients, information on other nutrients and the energy content of the product should be included in the nutrition declaration.

In Peru, although there is a technical standard that specifies the characteristics and how the nutritional information of beverages and foods should be declared, it is only optional for all packaged products sold in the Peruvian market ^7^. One of the exceptions is the declaration of trans fat content, whose current regulation states that all products with trans fat must declare so on their label ^8^. Available evidence reveals that, in 2018, before the implementation of octagons, 23.9% (n=657) of a sample of 2748 beverages and packaged foods offered in a supermarket chain in Lima, did not declare nutrient content, nor energy content on their packaging ^9^.

The mandatory declaration of nutritional information is an important tool for consumers to obtain complete and detailed information on the nutrient content, in order to make better informed decisions ^10^, as well as to monitor compliance with regulations related to the nutritional content of foods. In addition, this information allows corroborating the veracity of the nutritional or health claims included in the labeling ^11^. With regard to the information to be declared, the Codex Alimentarius states that nutritional information should indicate the energy value, content of macronutrients such as fats, proteins and carbohydrates, and particularly, saturated fats, trans fats, sugar and dietary fiber, as well as micronutrients such as vitamins and minerals, including sodium ^12^.

Following the implementation of new policies regarding nutrition labeling ^13^, our study aimed to estimate the amount of processed and ultra-processed foods and beverages offered in a supermarket chain in Lima in 2022 that declare nutritional information on the packaging as well as to describe their characteristics and determine the presence of nutritional information on products with octagons.

**KEY MESSAGES**

Motivation for the study. Peruvian Law No. 30021 establishes the use of warning octagons for foods with high content of critical nutrients (sugar, sodium, saturated and trans fats); however, the declaration of nutritional information is not mandatory.

Main findings. Of a total of 4404 processed and ultra-processed foods marketed in supermarkets in Lima, only 71.4% declared some type of nutritional information. In addition, only 46.0% declared information on the content of critical nutrients regulated by Law No. 30021.

Implications. There is a need for a mandatory and standardized declaration of nutritional information on packaged foods marketed in Peru, in order to allow the population to make healthy decisions when choosing their food and to monitor the correct use of warning octagons.

## MATERIALS AND METHODS

### Design

We carried out a descriptive cross-sectional study.

### Sampling and data collection

We collected information from the labels of all processed and ultra-processed foods and beverages with barcodes offered in the selected stores between May and June 2022. Information was collected from three stores of a supermarket chain located in Metropolitan Lima targeted at different socioeconomic levels, seeking to capture the widest possible variety of products. The collection began in the store with the largest number and variety of products, in the next store only the products that were not found in the first store were collected, and in the last one, we collected information about the products that were not found in the first two stores.

### Procedures

A team of eight previously trained field workers collected data on all processed and ultra-processed foods and beverages with barcodes, excluding fresh foods, minimally processed foods, ingredients (foods or beverages used in culinary preparations), alcoholic beverages and multiple packages (packages containing two or more different products). We used the FLIP application to register the products ^14^, which was developed by the University of Toronto, Canada. This application, after being installed on a cell phone, scans the barcodes to create an identification code (ID), as well as taking photographs of each side of the package and storing them directly in the system. FLIP is a paid application, after coordination with its developers.

Each of the photos was reviewed at the end of every day of data collection, verifying that the information was legible. Subsequently, the following data were entered into the web version of FLIP: name of the product, producing company, product description, among other characteristics that allow identifying the product, list of ingredients, nutritional information, presence of octagons. Products were classified into two categories: a) food, when the net weight in grams was declared, and b) beverages, when the net weight in milliliters was declared. These categories distributed into subcategories as detailed in Table 1.

**Table 1:** Categories and subcategories of processed and ultra-processed foods and beverages offered in a supermarket chain in Metropolitan Lima, in 2022.

Category Subcategory		Examples
1. Beverages		
	1.1. Artificial juices and nectars	Artificial juices and nectars
	1.2. Refreshments	Flavored and bottled beverages (bottled chicha morada, bottled orange juice)
	1.3. Carbonated beverages	Sodas with/without sugar, light or regular
	1.4. Sports beverages	Rehydrating beverages
	1.5. Energizing beverages	Energizing beverages
	1.6. Coffee-based beverages	Ultra-processed coffee-based beverages
	1.7. Infusions	Ready-to-drink infusions
	1.8. Milk	100% whole, skimmed, UHT, evaporated milk
	1.9. Milk-based beverages	Dairy mixes, drinkable yoghurt, flavored milk, milk-based beverages
	1.10. Milk substitutes	Beverages made from coconut, soy, almonds, among other grains.
	1.11. Formulas and dietary supplements	Formulas
	1.12. Soluble powders	Soluble powders for flavoring milk or water
	1.13. Chocolate bars/tablets	Chocolate bar for drinking
2. Food		
	2.1. Bread and bakery/pastry products	Packaged breads, tortillas, cookies, packaged cakes, panettone, etc.
	2.2. Cereals	Breakfast cereals, cereal bars, oatmeal with/without flavorings
	2.3. Sweets and desserts	Ice creams, chocolates, candies, gummies, marshmallows, jellies, custards
	2.4. Ready-to-eat meals	Instant soups, frozen, refrigerated and non-refrigerated ready meals, compotes for babies
	2.5. Meat, by-products and eggs	Processed meat (ham, , hot dogs, nuggets, hamburgers), dressed meats, powdered egg whites
	2.6. Fish and seafood	Canned fish, fish patties and nuggets, frozen seasoned seafood
	2.7. Dairy	Cheese, yogurt, cream milk, condensed milk
	2.8. Canned fruits and vegetables	Canned fruits, canned vegetables, canned olives, canned fruits and vegetables with frozen additives
	2.9. Sauces and spreads	Mayonnaise, mustard, ketchup, chili sauces, salad dressings, sweet spreads (jams, jelly, spreads, toppings), nut butters
	2.10. Salty snacks	Potato chips, corn tortillas, extruded snacks, nuts and seeds, chifles, pop corn
	2.11. Canned and preserved legumes	Dressed legumes, canned legumes
	2.12. Condiments and spices	Ready-made dressings, artificial seasonings, breading mixes, breading mixes
	2.13. Oils, butters and margarines	Butter
	2.14. Meat substitutes and dairy substitutes	Tofu, protein foods based on soybeans, grains or legumes, non-dairy spreads

UHT: Ultra Heat Treatment.

Once data was collected, the nutrient content was standardized to 100 g or 100 ml, including foods that require reconstitution, i.e., those products that require the addition of one or more ingredients to be consumed. We verified the quality of the data by identifying duplicate records using bar codes and comparing the data of all the records that did not comply with the Atwater validation ^15^ with the corresponding photographs.

The Atwater validation consists of multiplying the macronutrient content (total carbohydrate, total fat, protein) by the corresponding Atwater factor (carbohydrate*4 + protein*4 + fat*9) and the sum of these products must coincide with the total energy, accepting a range of ± 20%.

### Variables

We included the following variables:

- a. Foods and beverages that declare nutritional information (nutrient information or energy content must be declared).
- Foods and beverages that declare the energy content.
- Foods and beverages that declare information on the content of the three macronutrients (carbohydrates, total fats and proteins).
- Foods and beverages that declare information on the content of the four critical nutrients (sugar, sodium, saturated fat and trans fat).
- b. Characteristics of the nutritional information declared on the label.
- Format in which the nutritional information is declared (in table, text or other).
- Unit of measurement in which the nutrition information is declared (per serving, per 100 g or ml, in percent daily value).
- Consistency in the declaration of nutrition information that does not comply with the Atwater validation ^15^.
- Presence of nutrition information on foods with octagons.
- Foods and beverages with octagons.
- Foods and beverages that declare the nutritional information of the critical nutrient warned in the octagon.

In addition, we included information regarding the origin of the beverage or food, including the country where it was produced. Beverages and foods produced in Peru were classified as “national” and those produced in other countries as “imported”.

### Data analysis

We carried out a univariate analysis to determine the absolute and relative frequencies of each of the variables of interest (foods and beverages declaring nutritional information, characteristics of the nutritional information declared in the labeling, and presence of nutritional information in foods and beverages with octagons). The analyses were performed in Stata version 15 (StataCorp, College Station, TX, USA) was.

### Ethical criteria

The protocol was exempted from review by the Institutional Ethics Committee of the Universidad Peruana Cayetano Heredia (CAREG-ORVEI-016-22). Information regarding the supermarkets where the study was carried out will be provided upon request.

## RESULTS

A total of 4404 packaged products were analyzed, of which 81.0% (n=3579) were categorized as solid foods and 19.0% (n=825) as beverages. Of the total products, 90.7% (n=3996) were ready-to-eat, while 9.3% (n=408) required reconstitution.

We found that 71.8% (n=3161) of the products declared some nutritional information, and only 46.0% (n=2026) declared information on the four critical nutrients prioritized in the Peruvian regulation. The proportion of products declaring nutritional information for each category and subcategory is shown in Table 2.

**Table 2:** Processed and ultra-processed foods and beverages offered in a supermarket chain in Metropolitan Lima that declare nutritional information in 2022.

Packaged products		n (%)	Declares some nutritional information		Declares energy content information		Declares information on the three macronutrients		Declares information on the four critical nutrients	
			n	%	n	%	n	%	n	%
Beverages		825 (19)	761	92.2	744	90.2	706	85.6	385	46.7
	Artificial juices and nectars	114 (2.6)	89	78.1	89	78.1	86	75.4	29	25.4
	Refreshments	78 (1.8)	75	96.2	75	96.2	71	91.0	9	11.5
	Carbonated beverages	128 (2.9)	115	89.8	115	89.8	74	57.8	2	1.6
	Sports beverages	29 (0.7)	29	100.0	29	100.0	29	100.0	0	0.0
	Energizing beverages	16 (0.4)	16	100.0	16	100.0	11	68.8	1	6.3
	Coffee-based beverages	8 (0.2)	8	100.0	8	100.0	8	100.0	6	75.0
	Infusions	9 (0.2)	9	100.0	9	100.0	7	77.8	1	11.1
	Milk	71 (1.6)	67	94.4	60	84.5	67	94.4	57	80.3
	Milk beverages	157 (3.6)	146	93.0	141	89.8	146	93.0	136	86.6
	Dairy substitutes	42 (0.1)	42	100.0	40	95.2	2	100.0	32	76.2
	Formulas and food supplements	100 (2.3)	100	100.0	100	100.0	100	100.0	56	56.0
	Soluble powders	61 (1.4)	54	88.5	51	83.6	54	88.5	45	73.8
	Chocolate bars/tablets	12 (0.3)	11	91.7	11	91.7	11	91.7	11	91.7
Solid food		3579 (81.0)	2400	67.1	2372	66.3	2311	64.6	1641	45.9
	Bread and bakery/pastry products	599 (13.6)	401	66.9	400	66.8	398	66.4	338	56.4
	Cereals	245 (5.6)	200	81.6	199	81.2	188	76.7	169	69.0
	Sweets and desserts	630 (14.3)	533	84.6	532	84.4	517	82.1	349	55.4
	Ready-to-eat prepared meals	339 (7.7)	164	48.4	158	46.6	160	47.2	115	33.9
	Meat, by-products and eggs	350 (7.9)	133	38.0	127	36.3	118	33.7	69	19.7
	Fish and seafood	79 (1.8)	68	86.1	68	86.1	66	83.5	38	48.1
	Dairy	284(6.4)	172	60.6	163	57.4	154	54.2	97	34.2
	Canned fruits and vegetables	148 (3.4)	80	54.1	80	54.1	79	53.4	40	27.0
	Sauces and spreads	427 (9.7)	326	76.3	326	76.3	316	74.0	197	46.1
	Salty snacks	271 (6.2)	161	59.4	161	59.4	161	59.4	128	47.2
	Canned and preserved legumes	8 (0.2)	8	100.0	8	100.0	8	100.0	7	87.5
	Condiments and spices	108 (2.5)	70	64.8	66	61.1	70	64.8	36	33.3
	Oils, butters and margarines	55 (1.2)	49	89.1	49	89.1	43	78.2	35	63.6
	Meat substitutes and dairy substitutes	36 (0.8)	35	97.2	35	97.2	33	91.7	23	63.9
Total		4404 (100.0)	3161	71.8	3116	70.8	3017	68.5	2026	46.0

Of the total number of beverages and foods that reported information regarding its origin(n=4402), 26.9% (n=1186) were imported and 73.1% (n=3216) were national. We found that 86.3% (n=1023) of the total imported beverages and foods reported nutritional information, while only 66.4% (n=2137) of the total national products (n=2137) did so. However, the proportion of beverages and foods declaring information on the four prioritized critical nutrients is similar between imported (45.1%) and national (46.3%) products (Table 3).

**Table 3:** Beverages and processed and ultra-processed foods marketed in a supermarket chain in Metropolitan Lima that declare nutritional information, according to their place of origin.

Packaged products that declare their origin (n=4402)		n (%)	Declares some nutritional information		Declares energy content information		Declares information on the three macronutrients		Declares information on the four critical nutrients	
			n	%	n	%	n	%	n	%
Imported		1186 (26.9)	1023	86.3	1016	85.7	988	83.3	535	45.1
	Beverages	189 (15.9)	181	95.8	177	93.7	176	93.1	86	45.5
	Solid food	997 (84.1)	842	84.5	839	84.2	840	84.3	449	45.0
National		3216 (73.1)	2137	66.5	2099	65.3	2028	63.1	1490	46.3
	Beverages	636 (19.8)	580	91.2	567	89.2	530	83.3	299	47.0
	Solid food	2580 (80.2)	1557	60.3	1532	59.4	1498	58.1	1191	46.2

The subcategories of solid foods with the lowest proportion of products declaring some nutritional information were meats, meat products and eggs (38.0%) followed by ready-to-eat prepared meals (48.4%), canned fruits and vegetables (54.1%), snacks (59.4%) and condiments and dry spices (64.8%). Artificial juices and nectars (78.1%) were the least reported beverages.

The nutritional information reported on each food or beverage is presented in different ways. Of the total of 3161 products that declared information, 85.3% used tables, 13.8% used text and 0.9% used other forms, such as images (Supplementary Material 1).

Regarding units of measurement, 84.2% of the 3161 products declared its contents per serving, while 56.3% per 100 grams or 100 milliliters. In addition, 73.7% stated the percentage daily value.

Some the products did not maintain consistency between the amount of energy and macronutrients declared, in this regard we found that of the 2992 products declaring energy and macronutrients, 166 products (5.4%) did not comply with the Atwater validation (Table 4), the most frequent error being the lack of consistency between the total amount of kilocalories with the caloric intake of each nutrient. Examples of this error can be found in Supplementary Material 2.

**Table 4:** Characteristics of processed and ultra-processed foods and beverages that declare nutritional information in a supermarket chain in Metropolitan Lima.

		Beverages		Solid food		Total	
		n	%	n	%	n	%
Declared nutritional information		761	24.1	2400	75.9	3161	100
	As a table	668	21.1	2029	64.2	2697	85.3
	As text	79	2.5	358	11.3	437	13.8
	In other formats	14	0.4	13	0.4	27	0.9
Declared nutritional information		761	24.1	2400	75.9	3161	100
	Information per serving	621	19.6	2041	64.6	2662	84.2
	Information per 100 grams or milliliters	451	14.3	1328	42.0	1779	56.3
	Information in percent of daily value	532	16.8	1798	56.9	2330	73.7
Declared energy and the three macronutrients		689	23.0	2303	77.0	2992	100
Did not meet Atwater validation		25	0.8	141	4.7	166	5.4

On the other hand, of the total of 4404 beverages and foods collected, 55% (n=2431) contained at least one frontal advertising warning (octagons) on the label. Table 5 details the number of foods and beverages with octagons according to each of the four critical nutrients, and how many claim nutritional information for the nutrient warned in the octagon. Of the total number of foods with the octagon for trans fats (n=52), only 19.2% declared information on trans fat content, while of the total with the octagon for sodium (n=988), only 49.7% declare the sodium content on the package.

**Table 5:** Proportion of processed and ultra-processed foods and beverages marketed in a supermarket chain in Metropolitan Lima that contain label warnings (octagons) and that declare nutritional information of the nutrient warned.

			Beverages		Solid food		Total	
			n	%	n	%	n	%
Total beverages and foods			825	18.7	3579	81.3	4404	100.0
	Contained some octagon		159	19.3	2272	63.5	2431	55.0
	Had “High sugar” octagon		155	12.0	1136	88.0	1291	100.0
		Declared sugar content	109	70.3	793	69.8	902	69.9
	Had “High in saturated fat” octagon		8	0.6	1323	99.4	1331	100.0
		Declared saturated fat content	6	75.0	823	62.2	829	62.3
	Had “High sodium” octagon.		4	0.4	984	99.6	988	100.0
		Declared sodium content	3	75.0	488	49.6	491	49.7
	Had “trans fats” octagon		0	0.0	52	100.0	52	100.0
		Declared trans fat content	0	0.0	10	19.2	10	19.2

## DISCUSSION

The Food and Agriculture Organization of the United Nations (FAO) and the World Health Organization (WHO) have published normative documents to encourage countries to take actions aimed at educating the population on healthy food choices ^10^^,^^16^. These documents state that the nutritional information on the packages should include the energy value, macronutrients, nutrients such as saturated fats, trans fats, and dietary fiber, as well as micronutrients (vitamins and minerals). However, our findings reveal that only 71.4% of beverages and processed and ultra-processed foods declared some nutritional information, 70.8% declare the energy value and only 68.5% declared information regarding the three main macronutrients (carbohydrates, proteins and fats), all of which could be due to the fact that the declaration of nutritional information is not mandatory in Peru ^7^.

There have been several attempts to make nutrition labeling mandatory and standardized in Peru. The proposed amendments to Law No. 30021 ^17^ that did not materialize. The declaration of nutritional information is mandatory and standardized in most countries from the European Union and Mercosur, as well as in the United States of America, Chile, Colombia, Ecuador, Mexico, among others ^18^^,^^19^.

Mandatory and standardized nutrition information provides consumers with clear and reliable information on the nutritional content of packaged foods ^10^.

Studies from Europe and the United States on young adults with access to higher education found that the use of nutrition information on packaging was associated with healthier diets, i.e., with a lower intake of fat, sugar and sodium ^20^; and with attitudes towards healthier food choices ^21^. Thus, the promotion of the use and correct interpretation of nutritional information increases consumers’ knowledge about nutrition and, consequently, favors healthier choices ^10^^,^^22^. However, in Peru, a study with almost 300 university students found that, although 79% understand it, only 4% (n=12) participants checked the nutrition labeling ^23^. A first step in order to strengthen the use of nutrition labeling is the mandatory declaration of nutritional information on all packaged foods and beverages marketed in Peru, as well as by using informative campaigns to stimulate its comprehension and correct interpretation when buying said products.

Our results show that nutritional information in Peru is declared in different ways and by using different units of measurement. The most common unit of measurement is the serving size, which, according to previous studies, influences consumers when choosing the portion size; larger serving sizes can induce people to eat more and therefore increase the energy intake ^24^. Other studies have suggested that serving size claims may confuse consumers, who may interpret the declared serving size as the one they regularly consume ^18^. In addition, one study concluded that it is important to standardize serving size in Peru, since information per serving allows a better estimation of the amount of energy and nutrients consumed ^25^. On the other hand, another study stated that nutritional information should be declared per 100g or ml, because it allows comparison of energy and nutrient content ^26^. Thus, although there is evidence in favor of both measures, there is consensus on the importance of standardizing the declaration of nutritional information for better understanding.

The declaration of nutritional information, in addition to being mandatory and standardized, must include reliable information for the consumer. The fact that 5.5% of the products analyzed did not comply with the Atwater validation shows that the nutritional information described on their labels could be incorrect and, consequently, confuse or misinform the consumer. In this regard, a Chilean study found that 9.6% (n=92) of the products that declared nutritional information had some type of inaccuracy or error, the most common being the lack of consistency between the amount of partial fat in relation to total fat and the total calories versus the caloric intake of each nutrient ^27^. We found these same errors during our study, which reveals the importance of having systems that guarantee the accuracy and veracity of the information declared on packaged products.

As part of the implementation of the front-of-package warnings, the Peruvian government implemented the “*Julieta checa la etiqueta*” campaign aimed at promoting the reading and understanding of the octagons, as well as informing about the characteristics of the labels. However, this campaign did not mention that the information about the critical nutrient on the octagon could be found in the nutritional declaration ^28^, which could have increased the use of nutrition labeling. The non-inclusion of the aforementioned statement in this campaign may have been due to the fact that a significant number of products with octagons do not declare the amount of the nutrient found in the octagon, which prevents the consumer from having information on the exact content of that nutrient and potentially reducing the impact sought by the labeling warnings.

In addition, this lack of information, as well as the lack of accuracy and truthfulness in nutrition labeling, prevents the competent entities from adequately monitoring the use of octagons and its effects, such as the reformulation of processed and ultra-processed beverages and foods by the industry ^29^.

We found that only 19.2% of the products with the trans fats octagon declared their content on their labeling, despite the regulation indicating that all packaged products containing trans fats must declare so ^8^. In addition, the National Institute for the Defense of Competition and Protection of Intellectual Property (INDECOPI) issued a resolution authorizing that products with less than 0.5 g of trans fat per serving may declare them as zero (0) in their nutritional statement, based on the Food and Drug Administration (FDA) standard of the United States of America, instead of making the Peruvian standard prevail, thus allowing the industry to avoid the octagon ^30^. INDECOPI’s resolution directly affects the consumer, since products with trans fats may be hidden from consumers, giving untruthful information of their nutritional composition. Therefore, it is important to have a public policy that frames the regulations related to nutrition labeling in order to maintain consistency between them and ensure that all packaged products have standard, clear, complete and truthful nutritional information. In this way, consumers can make informed decisions regarding the foods they purchase and thus contribute to protecting the health of children and adolescents, who are exposed to beverages and foods with high contents of sugar, sodium and fat in the school environment ^(^^31^.

Our study has some strengths, such as the large number of beverages and ultra-processed foods included in the analysis, as well as the fact that the information was recent, since data collection was carried out after the second phase of the octagons implementation. One of the limitations of our study is that we collected information from products available in three supermarkets in Lima, but retail stores, convenience stores or markets, and common food outlets in Peru were excluded, which means that we could not analyze every beverage and packaged food.

In conclusion, slightly more than a quarter of the products evaluated in this study did not declare any nutritional information. Those that do declare this type of information do so in different ways, and 5.5% did not do so in a clear or reliable manner. A significant number of products with octagons, particularly those containing trans fats and those high in sodium, did not declare the amount of the nutrient on the octagon, which does not complement the information provided by the octagons in case the consumer or the regulatory institution requires it. These results reveal the need for a mandatory and standardized declaration of nutritional information on packaged foods marketed in Peru.
