# Supplementary material for: Nutritional information on the labels of processed and ultra-processed foods and beverages marketed in a supermarket chain in Lima in 2022
Source: Rev Peru Med Exp Salud Publica. 2023 Jun 30;40(2):141–9. doi: 10.17843/rpmesp.2023.402.12714 (PMC10953672; doi:10.17843/rpmesp.2023.402.12714)
Supplement: Supplementary material. — Available in the electronic version of the RPMESP. [file rpmesp-40-02-12714-s001.docx]

Suplementario 1

**Formatos en que se declara la información nutricional**

1. En tabla


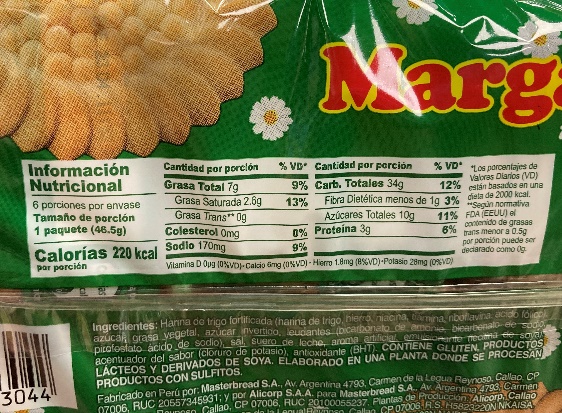


1. En texto


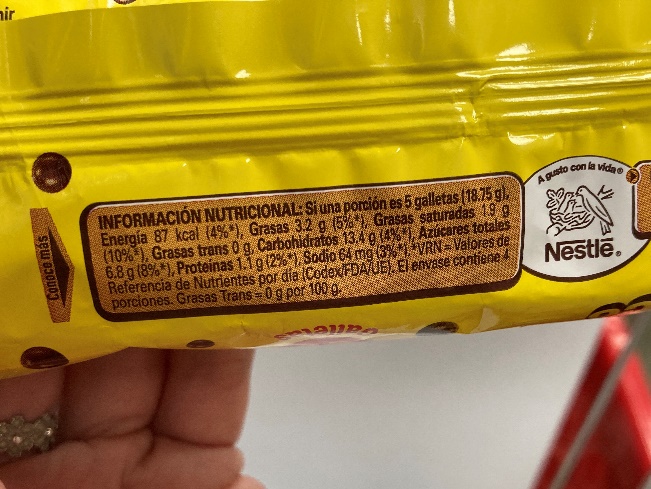


1. En imágenes


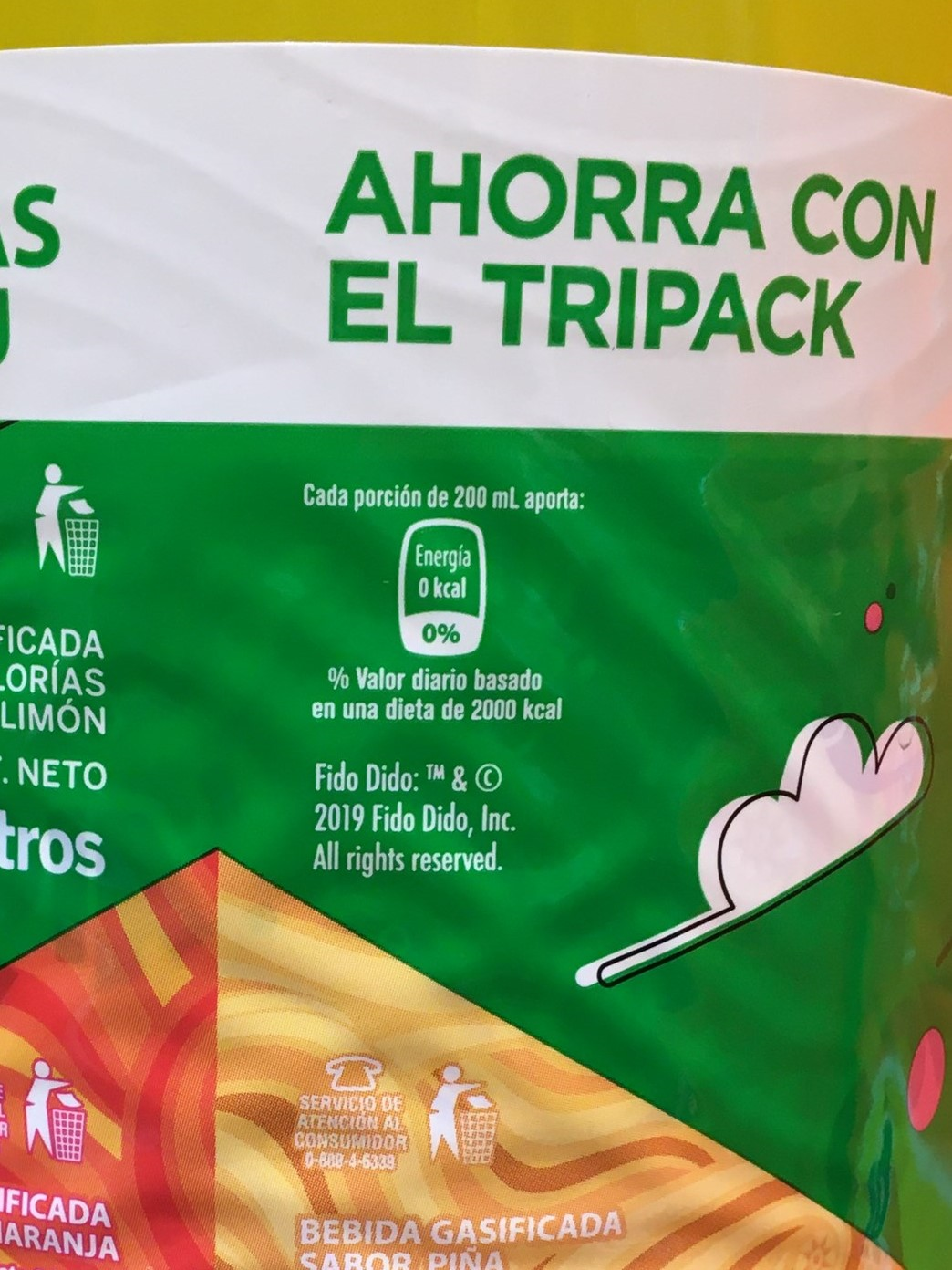

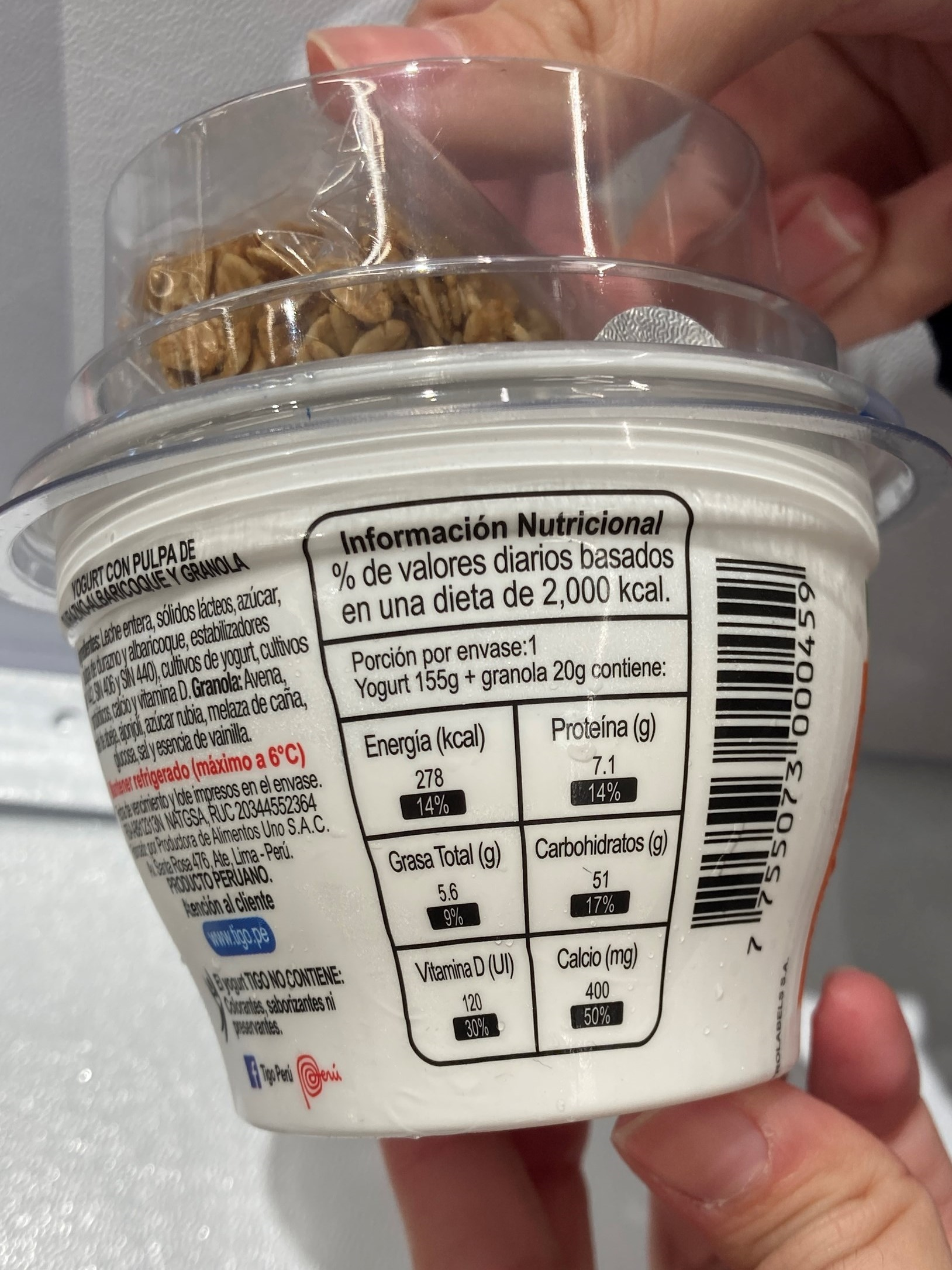


Suplementario 2

**Ejemplos de errores en la declaración de nutrientes**

1. Falta de coherencia entre la declaración de Energía y el aporte calórico de cada nutriente


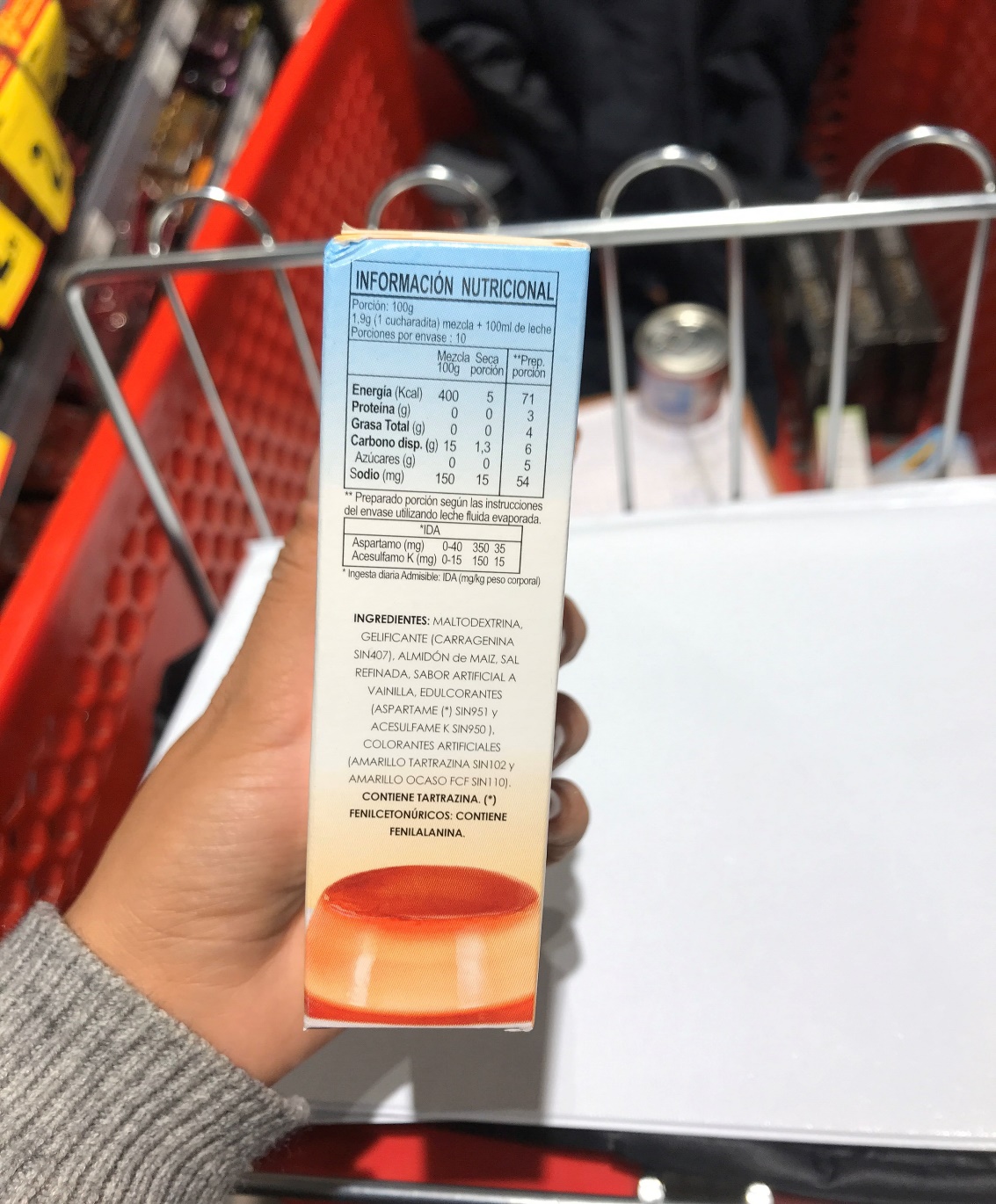

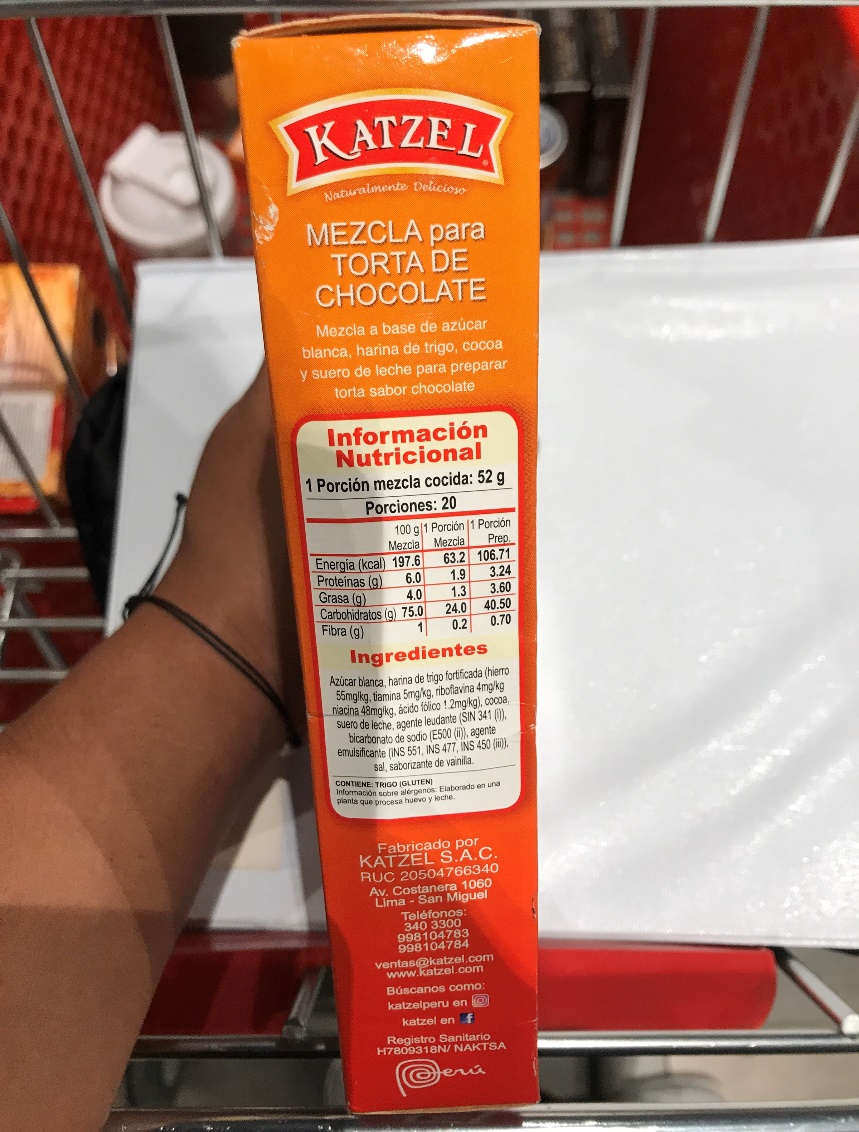


El aporte energético basado en la cantidad de los macronutrientes debe ser 60 kcal, sin embargo, el etiquetado declara 400 Kcal

El aporte energético basado en la cantidad de los macronutrientes debe ser 360 kcal, sin embargo, el etiquetado declara 197.6 Kcal

1. Incoherencia entre los carbohidratos totales y carbohidratos parciales


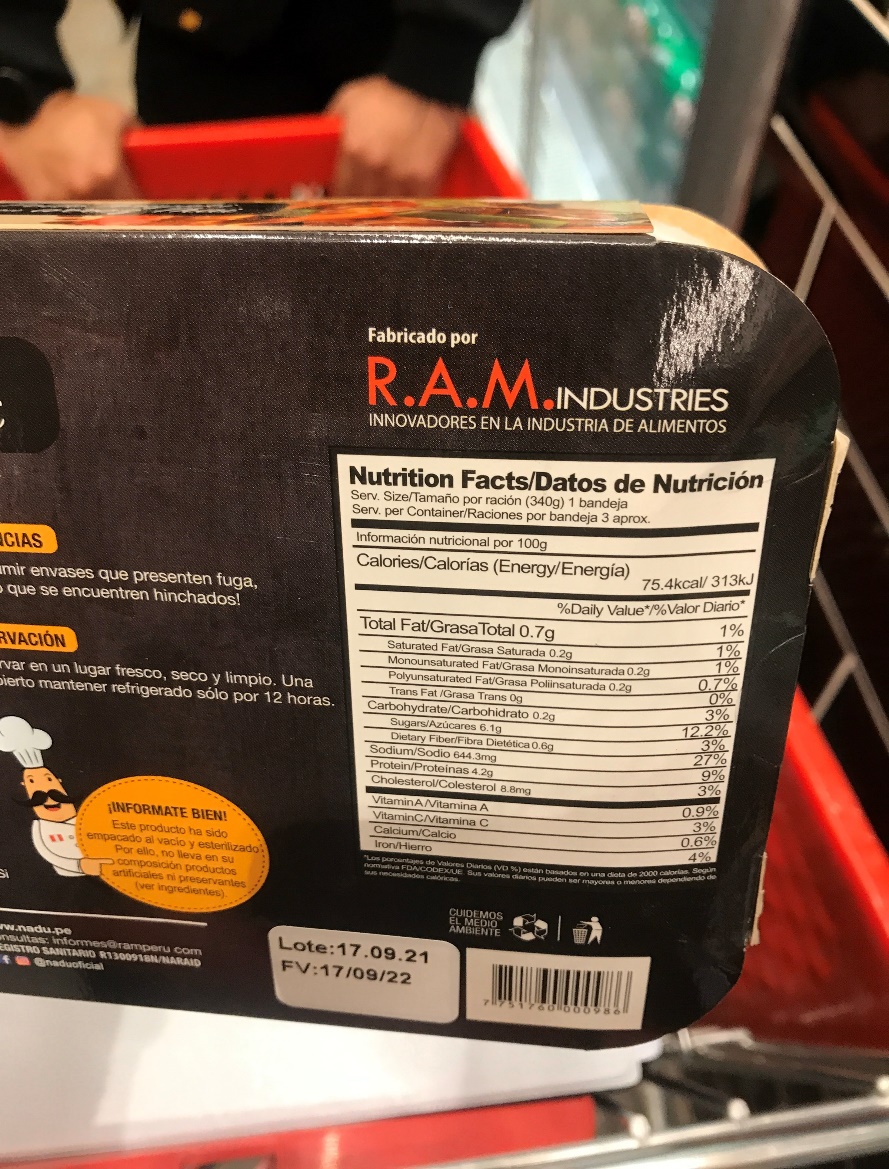


La cantidad de azúcares es superior a la cantidad de Carbohidratos
