# Supplementary material for: Nutritional information on the labels of processed and ultra-processed foods and beverages marketed in a supermarket chain in Lima in 2022
Source: Rev Peru Med Exp Salud Publica. 2023 Jun 30;40(2):141–9. doi: 10.17843/rpmesp.2023.402.12714 (PMC10953672; doi:10.17843/rpmesp.2023.402.12714)
Supplement: Translation

# Translation: Declaración de información nutricional en el etiquetado de bebidas y alimentos procesados y ultraprocesados ofertados en una cadena de supermercados de Lima en el 2022

**Authors:** Mayra Meza-Hernández, Kiomi Yabiku-Soto, Lorena Saavedra-Garcia, Francisco Diez-Canseco

**Affiliations:** 1 CRONICAS Centro de Excelencia en Enfermedades Crónicas, Universidad Peruana Cayetano Heredia, Lima, Perú.

**Keywords:** Etiquetado Nutricional, Información Nutricional, Alimentos Industrializados, Perú

## Abstract

**Objetivos.:**

Estimar la cantidad de bebidas y alimentos procesados y ultraprocesados que declaran información nutricional en su empaque y describir las características de esta información, así como determinar la presencia de información nutricional en los productos con octógonos.

**Materiales y métodos.:**

Se tomaron fotografías del etiquetado de 4404 bebidas y alimentos procesados y ultraprocesados ofertados en supermercados de Lima Metropolitana. La información declarada en la etiqueta se recogió y se registró en la versión móvil y web del Programa de Información de Etiquetas de Alimentos (FLIP por sus siglas en inglés). Se analizaron las variables correspondientes a la declaración de información nutricional, la forma en que se declara dicha información y la declaración de la misma en bebidas y alimentos con octógonos.

**Resultados.:**

De todos los productos recolectados solo el 71,4% declara algún tipo de información nutricional. De estos, el 13,8% declara la información nutricional en texto y no en tabla, además sólo 56,3% la declara por cada 100 gramos o mililitros. Del total de alimentos con el octógono «Contiene grasas trans», sólo 19,2% declara su contenido.

**Conclusiones.:**

Más de la cuarta parte de bebidas y alimentos envasados expendidos en el mercado peruano no declaran información nutricional de ningún tipo, y de los que declaran, existe una que lo hace en diferentes formatos y unidades. Además, se halló que hay una proporción de bebidas y alimentos para cada tipo de octógono que no declaran la información del nutriente advertido en el mismo.

## INTRODUCCIÓN

En los últimos años se ha incrementado el consumo de bebidas y alimentos procesados y ultraprocesados a nivel mundial ^1^. Este tipo de productos se caracteriza por contener elevadas cantidades de nutrientes críticos (grasas saturadas, azúcar, sodio y grasas trans), cuyo consumo en exceso es un factor de riesgo para desarrollar enfermedades crónicas no transmisibles ^2^. En el Perú, entre los años 2009 y 2014, las ventas de alimentos ultraprocesados se incrementaron en 8,9% (de 294 a 320 kcal per cápita/día), mientras que las de bebidas ultraprocesadas se incrementaron en 6,7% (de 114 a 121 kcal per cápita/día) ^1^.

Ante esta situación, el Gobierno peruano estableció políticas que promueven la alimentación saludable ^3^^-^^5^. Una de estas políticas es la Ley de Promoción de la Alimentación Saludable en Niños, Niñas y Adolescentes (Ley n.º 30021) ^3^. Esta ley indica que las bebidas y alimentos procesados y ultraprocesados que sobrepasen los parámetros establecidos para los nutrientes críticos, deben llevar una advertencia publicitaria frontal en forma de octógonos negros (en adelante «octógonos»). Estos octógonos indican que los productos presentan alto contenido de sodio, azúcar y/o grasas saturadas, o que contienen grasas trans ^6^. Sin embargo, si el consumidor busca conocer el contenido exacto de dichos nutrientes, información sobre otros nutrientes o el contenido de energía del producto, debería recurrir a la declaración de información nutricional.

En el Perú, aunque existe la norma técnica que especifica las características de la declaración de la información nutricional de bebidas y alimentos, esta no es obligatoria sino de carácter facultativo para todos los productos envasados que se expenden en el mercado peruano ^7^. Una de las excepciones es la declaración de contenido de grasas trans, cuya normativa vigente menciona que todo producto con grasas trans debe declararlo en su etiqueta ^8^. La evidencia disponible revela que, en el 2018, antes de la implementación de los octógonos, el 23,9% (n=657) de una muestra de 2748 bebidas y alimentos envasados ofertados en una cadena de supermercados de Lima, no declaraban el contenido de nutrientes, ni de energía en su empaque ^9^.

La declaración obligatoria de la información nutricional es una herramienta importante para que los consumidores conozcan de manera completa y detallada el contenido de nutrientes, y tomen decisiones mejor informados ^10^, además de permitir el monitoreo del cumplimiento de las regulaciones relacionadas al contenido nutricional de los alimentos. Adicionalmente, esta información permite corroborar la veracidad de las declaraciones nutricionales o de salud incluidas en el etiquetado ^11^. En relación a la información que se debe declarar, el Codex Alimentarius señala que la información nutricional debe indicar el valor energético, macronutrientes como las grasas, proteínas y carbohidratos, y algunos de estos de manera específica como grasas saturadas, grasas trans, azúcar y fibra alimentaria, además de micronutrientes como vitaminas y minerales, incluyendo el sodio ^12^.

Tras la implementación de nuevas políticas referidas al etiquetado nutricional ^13^, el presente estudio buscó estimar la cantidad de bebidas y alimentos procesados y ultraprocesados, ofertados en una cadena de supermercados de Lima en el 2022, que declaran información nutricional en el empaque y describir sus características, así como determinar la presencia de información nutricional en los productos con octógonos.

**MENSAJES CLAVE**

Motivación para realizar el estudio. La Ley peruana n.° 30021, establece el uso de octógonos de advertencia para alimentos con alto contenido de nutrientes críticos (azúcar, sodio, grasas saturadas y trans); sin embargo, la declaración de la información nutricional no es obligatoria.

Principales hallazgos. De un total de 4404 alimentos procesados y ultraprocesados ofertados en supermercados de Lima solo el 71,4% declaraba algún tipo de información nutricional. Además, solo el 46,0% declara información del contenido de nutrientes críticos regulados en la Ley N° 30021.

Implicancias. Se necesita contar con una declaración obligatoria y estandarizada de la información nutricional en los alimentos envasados que se comercializan en el Perú que permita a la población tomar decisiones saludables cuando elige sus alimentos y monitorear el correcto uso de las advertencias frontales (octógonos).

## MATERIALES Y MÉTODOS

### Diseño

Se realizó un estudio descriptivo de corte transversal.

### Muestreo y recolección de datos

Entre mayo y junio del 2022, se recolectó información declarada en la etiqueta de todos los alimentos y bebidas procesados y ultraprocesados con códigos de barras ofertados en las tiendas seleccionadas. La recolección tuvo lugar en tres tiendas de una cadena de supermercados ubicadas en Lima Metropolitana y dirigidas a diferentes niveles socioeconómicos, buscando así capturar la mayor variedad de productos posibles. La recolección se inició en la tienda con mayor número y variedad de productos, en la siguiente tienda solo se recolectaron los productos que no fueron ubicados en la primera y en la última, los productos que no fueron ubicados en las dos primera tiendas.

### Procedimientos

Un equipo de ocho trabajadores de campo previamente capacitados realizó la recolección de datos de todos los alimentos y bebidas procesados y ultraprocesados con códigos de barras, excluyendo los alimentos frescos, mínimamente procesados, ingredientes (alimentos o bebidas empleados en preparaciones culinarias), bebidas alcohólicas y empaques múltiples (empaques que contienen dos o más productos diferentes). Para registrar los productos, se empleó el aplicativo FLIP ^14^, desarrollado por la Universidad de Toronto, Canadá, y que, tras instalarse en un teléfono móvil permite escanear los códigos de barras creando un código de identificación (ID), así como capturar fotografías de cada lado del empaque y almacenarlas directamente en el sistema. El uso de este aplicativo es pagado, previa coordinación con sus desarrolladores.

Finalizado cada día de recolección, se revisó cada una de las fotos ingresadas al sistema, verificando que la información sea legible. Posteriormente, en la versión web de FLIP se digitaron los siguientes datos: nombre del producto, compañía productora, descripción del producto, entre otras características que permiten identificar el producto, listado de ingredientes, información nutricional, presencia de octógonos. Al mismo tiempo, los productos fueron clasificados en dos categorías: a) alimentos, cuando declaraban el peso neto en gramos, y b) bebidas, cuando declaraban el peso neto en mililitros, y a su vez se distribuyeron en subcategorías tal y como se detalla en la Tabla 1.

**Tabla 1:** Categorías y subcategorías de las bebidas y alimentos procesados y ultraprocesados que se ofertan en una cadena de supermercados de Lima Metropolitana, en el 2022.

Categoría Subcategoría		Ejemplos
1. Bebidas		
	1.1. Jugos y néctares artificiales	Jugos y néctares artificiales
	1.2. Refrescos	Bebidas saborizadas y envasadas (chicha morada envasada, refresco de naranja envasado)
	1.3. Bebidas carbonatadas	Gaseosas con/sin azúcar, *light* o regular
	1.4. Bebidas para deportistas	Rehidratantes
	1.5. Bebidas energizantes	Energizantes
	1.6. Bebidas a base de café	Bebidas ultraprocesadas en base a café
	1.7. Infusiones	Infusiones listas para tomar
	1.8. Leche	100% leche entera, descremada, UHT, evaporada
	1.9. Bebidas lácteas	Mezclas lácteas, yogurt bebible, leche saborizada, bebidas en base a leche
	1.10. Sustitutos lácteos	Bebidas elaboradas a base de coco, soya, almendras, entre otros granos.
	1.11. Fórmulas y suplementos alimenticios	Fórmulas
	1.12. Polvos solubles	Polvos solubles para saborizar la leche o el agua
	1.13. Barras/tabletas/ pasta de chocolate	Chocolate para taza en barra
2. Alimentos		
	2.1. Pan y productos de panadería/pastelería	Panes envasados, tortillas, galletas, queques envasados, panetones, turrones
	2.2. Cereales	Cereales para desayuno, barras de cereal, avenas con/sin saborizantes
	2.3. Golosinas y postres	Helados, chocolates, caramelos, gomitas, *marshmellows*, gelatinas, flanes, mazamorras
	2.4. Comidas preparadas listas para comer	Sopas instantáneas, comida preparada congelada, refrigerada y sin refrigerar, compotas para bebés
	2.5. Carnes, derivados y huevos	Carne procesada (jamón, jamonada, *hot dog, nuggets*, hamburguesas), carnes aderezadas, clara de huevo en polvo
	2.6. Pescados y mariscos	Pescados en conserva, hamburguesas y *nuggets* de pescado, mariscos aderezados congelados
	2.7. Lácteos	Quesos, yogurt, crema de leche, leche condensada
	2.8. Frutas y verduras en conserva	Frutas en conserva, verduras en conserva, aceitunas en conserva, frutas y verduras con aditivos congeladas
	2.9. Salsas y untables	Mayonesa, mostaza, *ketchup*, salsas de ají, aderezos para ensaladas, untables dulces (mermeladas, manjar, coberturas), mantequilla de nueces
	2.10. Piqueos / snacks salados	Chips de papas, tortillas de maíz, snacks extruidos, nueces y semillas, chifles, *pop corn*
	2.11. Legumbres en conserva y enlatadas	Legumbres aderezadas, legumbres en conserva
	2.12. Condimentos y especias	Aderezos listos, sazonadores artificiales, mezclas para apanar
	2.13. Aceites, mantequillas y margarinas	Mantequillas
	2.14. Sustitutos de carne y sustitutos de lácteos	Tofu, alimentos proteicos a base de soya, granos o legumbres, cremas no lácteas

UHT: Ultra Heat Treatment (Tratamiento a altas temperaturas).

Una vez terminada la digitación, se estandarizó el contenido de nutrientes a 100 g o 100 ml, incluyendo los alimentos que requieren reconstitución, es decir, aquellos productos que necesitan agregar uno o más ingredientes para que puedan ser consumidos. Así también, se verificó la calidad de los datos digitados en la base, identificando registros duplicados a través de los códigos de barras y se compararon los datos de todos los registros que no cumplían con la validación Atwater ^15^ con las fotografías correspondientes, con el fin de corroborar que no se trataba de un error durante la digitación.

La validación Atwater consiste en multiplicar el contenido de los macronutrientes (carbohidratos totales, grasas totales, proteínas) por el factor Atwater correspondiente (carbohidrato*4 + proteína*4 + grasas*9) y la sumatoria de estos productos debe coincidir con el total de energía, aceptando un rango de ± 20%.

### Variables

Las variables incluidas en el presente estudio fueron:

- a. Alimentos y bebidas que declaran información nutricional, para ello deben declarar la información de un nutriente o del contenido de energía.
- Alimentos y bebidas que declaran el contenido de energía.
- Alimentos y bebidas que declaran información sobre el contenido de los tres macronutrientes (carbohidratos, grasas totales y proteínas).
- Alimentos y bebidas que declaran información sobre el contenido de los cuatro nutrientes críticos (azúcar, sodio, grasas saturadas y grasas trans).
- b. Características de la información nutricional declarada en el etiquetado.
- Formato en que se declara la información nutricional (en tabla, en texto u otro).
- Unidad de medida en la que se declara la información nutricional (por porción, por 100 g o ml, en porcentaje de valor diario).
- Coherencia en la declaración de la información nutricional que no cumple con la validación Atwater ^15^.
- Presencia de información nutricional en alimentos con octógonos.
- Alimentos y bebidas con octógonos.
- Alimentos y bebidas que declaran la información nutricional del nutriente crítico advertido en el octógono.

Además, se evalúo la presencia de etiquetado según la procedencia de la bebida o alimento, en la cual se identificó el país donde fue elaborado, a partir de lo declarado en la etiqueta. Una vez identificado el país, se clasifico como nacionales a las bebidas y alimentos elaborados en el Perú y como importados a los que fueron elaborados en otros países.

### Análisis de datos

Se realizaron análisis univariados para determinar las frecuencias absolutas y relativas de cada una de las variables de interés (alimentos y bebidas que declaran información nutricional, características de la información nutricional declarada en el etiquetado y presencia de información nutricional en los alimentos y bebidas con octógonos). Para ello se empleó el programa Stata versión 15 (StataCorp, College Station, TX, EUA). 

### Criterios éticos

El protocolo fue exonerado de revisión por el Comité Institucional de Ética de la Universidad Peruana Cayetano Heredia (CAREG-ORVEI-016-22). La información respecto a los supermercados donde se llevo a cabo el estudio se brindarán previa solicitud a los autores. 

## RESULTADOS

Se analizaron 4404 productos envasados, de los cuales el 81,0% (n=3579) fueron categorizados como alimentos sólidos y el 19,0% (n=825) como bebidas. Del total de productos, el 90,7% (n=3996) estaban listos para consumir, mientras que el 9,3% (n=408) requerían reconstitución.

En relación con la declaración de información nutricional, se encontró que el 71,8% (n=3161) declara alguna información nutricional, y sólo el 46,0% (n=2026) declara información sobre los cuatro nutrientes críticos priorizados en la regulación peruana. La proporción de productos que declarar información nutricional por cada categoría y subcategoría se puede observar en la Tabla 2.

**Tabla 2:** Bebidas y alimentos procesados y ultraprocesados ofertados en una cadena de supermercados de Lima Metropolitana que declaran información nutricional en el 2022.

Productos envasados		n (%)	Declara alguna información nutricional		Declara información del contenido de energía		Declara información de los tres macronutrientes		Declara información de los cuatro nutrientes críticos	
			n	%	n	%	n	%	n	%
Bebidas		825 (19)	761	92,2	744	90,2	706	85,6	385	46,7
	Jugos y néctares artificiales	114 (2,6)	89	78,1	89	78,1	86	75,4	29	25,4
	Refrescos	78 (1,8)	75	96,2	75	96,2	71	91,0	9	11,5
	Bebidas carbonatadas	128 (2,9)	115	89,8	115	89,8	74	57,8	2	1,6
	Bebidas para deportistas	29 (0,7)	29	100,0	29	100,0	29	100,0	0	0,0
	Bebidas de energizantes	16 (0,4)	16	100,0	16	100,0	11	68,8	1	6,3
	Bebidas a base de café	8 (0,2)	8	100,0	8	100,0	8	100,0	6	75,0
	Infusiones	9 (0,2)	9	100,0	9	100,0	7	77,8	1	11,1
	Leche	71 (1,6)	67	94,4	60	84,5	67	94,4	57	80,3
	Bebidas lácteas	157 (3,6)	146	93,0	141	89,8	146	93,0	136	86.6
	Sustitutos lácteos	42 (0,1)	42	100,0	40	95,2	2	100,0	32	76,2
	Formulas y suplementos alimenticios	100 (2,3)	100	100,0	100	100,0	100	100,0	56	56,0
	Polvos solubles	61 (1,4)	54	88,5	51	83,6	54	88,5	45	73,8
	Barras/tabletas/pastas de chocolate	12 (0,3)	11	91,7	11	91,7	11	91,7	11	91,7
Alimentos sólidos		3579 (81,0)	2400	67,1	2372	66,3	2311	64,6	1641	45,9
	Pan y productos de panadería/pastelería	599 (13,6)	401	66,9	400	66,8	398	66,4	338	56,4
	Cereales	245 (5,6)	200	81,6	199	81,2	188	76,7	169	69,0
	Golosinas y postres	630 (14,3)	533	84,6	532	84,4	517	82,1	349	55,4
	Comidas preparadas listas para comer	339 (7,7)	164	48,4	158	46,6	160	47,2	115	33,9
	Carnes, derivados y huevos	350 (7,9)	133	38,0	127	36,3	118	33,7	69	19,7
	Pescados y mariscos	79 (1,8)	68	86,1	68	86,1	66	83,5	38	48,1
	Lácteos	284(6,4)	172	60,6	163	57,4	154	54,2	97	34,2
	Frutas y verduras en conserva	148 (3,4)	80	54,1	80	54,1	79	53,4	40	27,0
	Salsas y untables	427 (9,7)	326	76,3	326	76,3	316	74,0	197	46,1
	Piqueos / snacks salados	271 (6,2)	161	59,4	161	59,4	161	59,4	128	47,2
	Legumbres en conserva y enlatadas	8 (0,2)	8	100,0	8	100,0	8	100,0	7	87,5
	Condimentos y especias	108 (2,5)	70	64,8	66	61,1	70	64,8	36	33,3
	Aceites, mantequillas y margarinas	55 (1,2)	49	89,1	49	89,1	43	78,2	35	63,6
	Sustitutos de carne y sustitutos de lácteos	36 (0,8)	35	97,2	35	97,2	33	91,7	23	63,9
Total		4404 (100,0)	3161	71,8	3116	70,8	3017	68,5	2026	46,0

En relación con la procedencia, del total de bebidas y alimentos que la reportan (n=4402) el 26,9% (n=1186) son importados y el 73,1% (n=3216) son nacionales. Se encontró que el 86,3% (n=1023) del total de bebidas y alimentos importados declara información nutricional, mientras que, del total de nacionales solo el 66,4% (n=2137) la declara. Sin embargo, la proporción de bebidas y alimentos que declaran información de los cuatro nutrientes críticos priorizados son similares entre los importados (45,1%) y nacionales (46,3%) (Tabla 3).

**Tabla 3:** Bebidas y alimentos procesados y ultraprocesados ofertados en una cadena de supermercados de Lima Metropolitana que declaran información nutricional, según su lugar de procedencia.

Productos envasados que declaran procedencia (n=4402)		n (%)	Declara alguna información nutricional		Declara información del contenido de energía		Declara información de los tres macronutrientes		Declara información de los cuatro nutrientes críticos	
			n	%	n	%	n	%	n	%
Importados		1186 (26,9)	1023	86,3	1016	85,7	988	83,3	535	45,1
	Bebidas	189 (15,9)	181	95,8	177	93,7	176	93,1	86	45,5
	Alimentos sólidos	997 (84,1)	842	84,5	839	84,2	840	84,3	449	45,0
Nacionales		3216 (73,1)	2137	66,5	2099	65,3	2028	63,1	1490	46,3
	Bebidas	636 (19,8)	580	91,2	567	89,2	530	83,3	299	47,0
	Alimentos sólidos	2580 (80,2)	1557	60,3	1532	59,4	1498	58,1	1191	46,2

Entre los alimentos sólidos, las subcategorías con menor proporción de productos que declaran alguna información nutricional son las carnes, derivados y huevos (38,0%) seguido de las comidas preparadas listas para comer (48,4%), las frutas y verduras en conserva (54,1%), los piqueos y snacks (59,4%) y los condimentos y especias secas (64,8%). Entre las bebidas, los jugos y néctares artificiales (78,1%) son los que menos declaran.

La información nutricional reportada en cada alimento o bebida se presenta en diferentes formatos. Del total de 3161 productos que declaran información, el 85,3% emplea una tabla, el 13,8% texto y el 0,9% utiliza otras formas, como imágenes (Material suplementario 1).

Respecto a la unidad de medida de la información nutricional, el 84,2% de los 3161 productos la declara por porción, mientras que el 56,3% por cada 100 gramos o 100 mililitros. Además, el 73,7% declara el porcentaje de valor diario.

En relación con la proporción de productos que no mantienen la coherencia entre la cantidad de energía y de macronutrientes declarados, se encontró que de los 2992 productos que declaran energía y macronutrientes, 166 productos (5,4%) no cumplen con la validación Atwater (Tabla 4), siendo el error más frecuente la falta de coherencia entre la cantidad total de kilocalorías con el aporte calórico de cada nutriente. Ejemplos de este error se puede ver en el Material suplementario 2.

**Tabla 4:** Características de las bebidas y alimentos procesados y ultraprocesados que declaran información nutricional en una cadena de supermercados de Lima Metropolitana.

		Bebidas		Alimentos sólidos		Total	
		n	%	n	%	n	%
Declaran información nutricional		761	24,1	2400	75,9	3161	100
	En tabla	668	21,1	2029	64,2	2697	85,3
	En texto	79	2,5	358	11,3	437	13,8
	En otros formatos	14	0,4	13	0,4	27	0,9
Declaran información nutricional		761	24,1	2400	75,9	3161	100
	Información por porción	621	19,6	2041	64,6	2662	84,2
	Información por cada 100 gramos o mililitros	451	14,3	1328	42,0	1779	56,3
	Información en porcentaje de valor diario	532	16,8	1798	56,9	2330	73,7
Declaran energía y los tres macronutrientes		689	23,0	2303	77,0	2992	100
No cumplen validación Atwater		25	0,8	141	4,7	166	5,4

Por otro lado, del total de 4404 bebidas y alimentos recolectados, el 55% (n=2431) contienen al menos una advertencia publicitaria frontal (octógonos) en la etiqueta. En la Tabla 5 se detalla la cantidad de alimentos y bebidas con octógonos según cada uno de los cuatro nutrientes críticos, y cuántos declaran información nutricional del nutriente advertido en el octógono. Del total de alimentos con el octógono para grasas trans (n=52), sólo el 19,2% declara información sobre el contenido de grasas trans, mientras que del total con el octógono para sodio (n=988) sólo el 49,7% declara el contenido de sodio en el envase.

**Tabla 5:** Proporción de alimentos y bebidas procesados y ultraprocesados ofertados en una cadena de supermercados de Lima Metropolitana que contienen advertencias publicitarias (octógono) y que declaran información nutricional del nutriente advertido.

			Bebidas		Alimentos sólidos		Total	
			n	%	n	%	n	%
Total de bebidas y alimentos recolectados			825	18,7	3579	81,3	4404	100,0
	Contiene algún octógono		159	19,3	2272	63,5	2431	55,0
	Contiene octógono «Alto en azúcar»		155	12,0	1136	88,0	1291	100,0
		Declara contenido de azúcar	109	70,3	793	69,8	902	69,9
	Contiene octógono «Alto en grasas saturadas»		8	0,6	1323	99,4	1331	100,0
		Declara contenido de grasas saturadas	6	75,0	823	62,2	829	62,3
	Contiene octógono «Alto en sodio»		4	0,4	984	99,6	988	100,0
		Declara contenido de sodio	3	75,0	488	49,6	491	49,7
	Contiene octógono «Contiene grasas trans»		0	0,0	52	100,0	52	100,0
		Declara contenido de grasas trans	0	0,0	10	19,2	10	19,2

## DISCUSIÓN

La Organización de las Naciones Unidas para la Alimentación y la Agricultura (FAO por sus siglas en inglés) y la Organización Mundial de la Salud (OMS) publicaron documentos normativos para promover que los países realicen acciones que eduquen a la población en la elección de alimentos saludables ^10^^,^^16^. Estos documentos indican que la declaración de información nutricional debería incluir el valor energético, macronutrientes, nutrientes como grasas saturadas, trans y fibra alimentaria, además de micronutrientes (vitaminas y minerales). Sin embargo, nuestros hallazgos revelan que de los productos evaluados solo el 71,4% de bebidas y alimentos procesados y ultraprocesados declaran alguna información nutricional, el 70,8% declaran el valor energético y únicamente 68,5% los tres macronutrientes principales (carbohidratos, proteínas y grasas), todo lo cual podría deberse a que la declaración de información nutricional no es obligatoria en el Perú ^7^.

En el Perú ha habido varios intentos para que el etiquetado nutricional sea obligatorio y estandarizado. Así lo evidencian las propuestas de modificatorias a la ley n.° 30021 ^17^ que no prosperaron. En países miembros de la Unión Europea, del Mercosur, así como en Estados Unidos de América, Chile, Colombia, Ecuador, México, entre otros; la declaración de la información nutricional es obligatoria y, en la mayoría de ellos, estandarizada ^18^^,^^19^.

La información nutricional obligatoria y estandarizada permite al consumidor contar con información clara y confiable sobre el contenido nutricional de los alimentos envasados ^10^.

Estudios en Europa y Estados Unidos con adultos jóvenes con acceso a la educación superior encontraron que el uso de la información nutricional de los empaques está asociado con la adopción de dietas más saludables, es decir, con una menor ingesta de grasas, azúcar y sodio ^(^^20^; y con actitudes dirigidas a la elección de alimentos más saludables ^21^. Es entonces que la promoción del uso y correcta interpretación de la información nutricional incrementa los conocimientos de los consumidores sobre nutrición y en consecuencia, favorece las decisiones más saludables ^10^^,^^22^. Sin embargo, en el Perú, un estudio con casi 300 estudiantes universitarios encontró que, aunque el 79% lo comprenden, solo el 4% (n=12) revisan siempre el etiquetado nutricional ^23^. Un primer paso para incrementar el uso del etiquetado nutricional es la declaración obligatoria de la información nutricional en todos los alimentos y bebidas envasados que se expenden en el mercado peruano, acompañado de campañas informativas que estimulen su lectura y correcta interpretación al tomar decisiones de compra.

Nuestros resultados muestran además que la declaración de la información nutricional en el Perú se realiza en diferentes formatos y unidades de medida. Entre las unidades de medida en que se declara la información nutricional, la más común es el tamaño de porción, el cual, según los estudios disponibles, influye en la elección de la de porción consumida; cuando se declaran tamaños de porción más grandes puede inducir a comer más cantidad y por ende en la ingesta de energía ^24^. Otros estudios sugieren que la declaración por tamaño de porción puede llegar a confundir al consumidor, quien podría interpretar el tamaño de porción declarado con el que consume regularmente ^18^. Además, una investigación concluyó que es relevante que en el Perú se estandarice el tamaño de porción, ya que la información por porción permite estimar mejor la cantidad de energía y nutrientes que se consume ^25^. Por otro lado, otro estudio menciona que es preferible que la información nutricional se declare por cada 100 g o ml, debido a que permite comparar el contenido de energía y nutrientes ^26^. Así, aunque existen evidencias en favor de ambas medidas, existe consenso en la importancia de la estandarización de la declaración de la información nutricional para su mejor comprensión.

La declaración de la información nutricional, además de obligatoria y estandarizada, debe ser confiable para el consumidor. Que el 5,5% de los productos analizados no cumpla con la validación Atwater revela que la información nutricional descrita en sus etiquetas podría ser incorrecta y en consecuencia, confundir o desinformar al consumidor. Al respecto, un estudio chileno encontró que en el 9,6% (n=92) de los productos que declaran información nutricional, se presenta algún tipo de inexactitud o error, siendo los más comunes la falta de coherencia entre la cantidad de grasas parciales con relación a las grasas totales y el total de calorías frente al aporte calórico de cada nutriente ^27^. Estos mismos errores se encontraron en nuestro estudio, lo que revela la importancia de contar con sistemas que garanticen la precisión y veracidad de la información declarada en los productos envasados.

En marco de la implementación de las advertencias publicitarias frontales, el Gobierno peruano implementó la campaña de «Julieta checa la etiqueta» teniendo como objetivo principal el promover la lectura y comprensión de los octógonos, así como informar sobre las características de las etiquetas. Sin embargo, dicha campaña no mencionaba que la información sobre el nutriente crítico advertido en el octógono podía encontrarse en la declaración de la información nutricional ^28^, lo cual hubiera podido incrementar el uso del etiquetado nutricional. La no inclusión del punto antes mencionado en dicha campaña se puede deber a que una proporción importante de productos con octógonos no declaran la cantidad del nutriente advertido en el mismo, impidiendo que el consumidor cuente con la información sobre el contenido exacto de dicho nutriente y, reduciendo potencialmente el impacto buscado por las advertencias publicitarias.

Además, esta ausencia de información como la falta de precisión y veracidad en el etiquetado nutricional impide que las entidades competentes monitoreen adecuadamente el cumplimiento del uso del octógono y sus efectos, como podría ser la reformulación de bebidas y alimentos procesados y ultraprocesados por parte de la industria ^29^.

A pesar de la reglamentación que indica que todos los productos envasados que contengan grasas trans deben declarar su contenido ^8^, este estudio encontró que solo el 19,2% de aquellos que tienen el octógono para grasas trans las declaran en su etiquetado. Además, el Instituto Nacional de Defensa de la Competencia y de la Protección de la Propiedad Intelectual (INDECOPI) emitió una resolución que autoriza que los productos que tengan menos de 0,5 g de grasas trans por porción podrán declararlas como cero (0) en su información nutricional, basándose en la norma de la Food and Drug Administration (FDA) de los Estados Unidos de América, en lugar de hacer prevalecer la norma peruana, permitiendo así que la industria pueda evitar el octógono ^30^. La resolución del INDECOPI afecta directamente al consumidor, ya que los productos con grasas trans pueden estar ocultos a los consumidores, dando una información poco verídica en cuanto a su composición nutricional. Por lo tanto, resulta de particular importancia la existencia de una política pública que enmarque las normativas relacionadas al etiquetado nutricional con el fin de mantener la consistencia entre ellas y garantizar que todos los productos envasados tengan información nutricional estándar, clara, completa y veraz. De esta manera, los consumidores pueden tomar decisiones informadas respecto a los alimentos que adquieren y así contribuir en la protección de la salud de niños, niñas y adolescentes, que están expuestos a bebidas y alimentos con alto contenido en azúcar, sodio y grasas en el entorno escolar ^31^.

Entre las fortalezas del presente estudio está la importante cantidad de bebidas y alimentos ultraprocesados incluidos en el análisis y la información reciente, ya que la recolección se realizó luego de la implementación de la segunda fase de los octógonos. Dentro de las limitaciones se puede mencionar que, si bien se recogieron todos los productos disponibles en tres supermercados de Lima, no se incluyeron tiendas minoristas, tiendas por conveniencia o mercados, puntos de venta de alimentos comunes en Perú, lo cual implica que no se ha analizado la totalidad de la oferta de bebidas y alimentos envasados.

En conclusión, un poco más de la cuarta parte de productos evaluados en el presente estudio no declaran ninguna información nutricional. Los que declaran lo hacen con distintos formatos y un 5,5% no lo hacen de forma clara y confiable. Una proporción importante de productos con octógonos, en particular los que contienen grasas trans y los altos en sodio, no declaran la cantidad del nutriente advertido, lo cual no permite complementar la información brindada por los octógonos en caso el consumidor o la institución reguladora lo requieran. Estos resultados revelan la necesidad de contar con una declaración obligatoria y estandarizada de la información nutricional en los alimentos envasados que se comercializan en el Perú.
